# The Making of the Modern Practitioner

**Published:** 1911-12-16

**Authors:** 


### THE MAKING OF THE MODERN PRACTITIONER.

### A New Series of Medical Text Books.

Among the items which contribute to the perfecting of the medical student—the practitioner in embryo—there are few which are more important than his books. Some four years ago, a series of monographs on special subjects was initiated under extremely able editorship by the firms of Hodder and Stoughton, and Henry Frowde, for the Oxford University Press. The series was known as the Oxford Medical Publications, and several of its components have been the subjects of review in the pages of The Hospital, as they appeared. The authors of this series were chiefly specialists, and the great majority of them had no connection with the University of Oxford. The same two firms now announce a new series, published by them for the University of London Press, Ltd., an organisation which is apparently bent on emulating the success of the volumes published for the Oxford University Press.

The name of the series is the "London Practitioners' Manuals," and the first two have now been published, while three more are in preparation. The general *format* is perhaps not quite the equal of the Oxford publications, and there are signs that the editing has not been quite so careful. Nevertheless a high standard is reached, and the publishers are to be congratulated on their continued enterprise. Apparently London University graduates and teachers are being mainly enlisted in the authorship, though they are, naturally, specialists, not general practitioners. Each volume is published at 6s. net.

### Minor Surgery.

This subject is entrusted to the capable pen of Mr. L. A. Bidwell, who has amplified for the purpose a course of his lectures at the West London Post-graduate College. It gives one a mild shock to find so experienced an author writing half his preface in the third, and the other half in the first, person; surely the newer University is not less jealous of its literary standard that the older one?

The author's advice on the various topics of which he treats may be followed implicitly; for it is throughout sane, practical, and full of common-sense. The subject of sutures is excellently discussed, and it is refreshing to see horsehair supported as a skin suture in minor surgery; but the subcuticular stitch, so useful in dealing with parts where a scar will be visible, is not mentioned. The holding power of the "granny" knot is stated to be equal to that of the "reef," an opinion which one is especially glad to see advanced, since most people cherish the contrary belief; the real advantage of the "reef" knot is, as Mr. Bidwell says, that it is more easily untied. Another sound observation is that about vaccines, which are available, the author notes, only for the very rich or the very poor; this too true observation confirms the contentions upheld in these columns not long ago (The Hospital, November 4, 1911, p. 118).

### Strychnine for Shock.

There are, however, one or two points in regard to which some dissent may be expressed. Strychnine, for instance, is recommended for shock on pp. 31 and 242; and denounced for the same condition on p. 47. The descriptions of the wool-skein truss and of the clove-hitch knot are impossible to understand unless the reader already knows how to make those two useful contrivances. Again, the following sentence from p. 46 mystifies one entirely: "An insufficient supply of blood to the skin prevents the temperature of the body being kept up and encourages shock." The author's decision to omit bandaging and the treatment of dislocations is excellent: the practitioner does not require the first, and if he wants information on the second he goes to a text-book of surgery. Why then should descriptions of operations for epulis, harelip, appendix abscess, and strangulated hernia be included, especially as "tonsils and adenoids" are not mentioned? Many pages, too, are devoted to the arrangement of an operating-room for major operations, which hardly falls within the proper scope of minor surgery.

### Anæsthesia and Analgesia.

There are already a half-dozen or more fairly recent monographs on this subject, and of approximately the same scope as this; so that an addition to the number must justify itself completely. The author, Dr. Mortimer, has on several occasions contributed to The Hospital, which is thus entitled to take an added interest in his volume. It may be fairly asserted that this maintains the standard of Mr. Bidwell's achievement, and is essentially of the nature of a practical guide to those pitfalls and difficulties which everywhere beset the path of the anæsthetist. One of the points which the author dwells upon appears to be novel—at least one does not remember to have seen it remarked before; this is that when post-anæsthetic vomiting is taking place, the jaw should not be pushed forward, as in so doing the larynx may be rendered patent and thus stomach contents may be drawn into the trachea. The author holds that circulatory shock is more likely to arise as a result of operative procedures during light than during deep anæsthesia: this is an interesting disputed point, and no evidence is adduced in support of the opinion expressed. On a kindred matter Mr. Mortimer is surely in a minority; for he believes that the prolongation of (anæsthesia for) an operation does not of itself render vomiting more likely. He also seemingly allows and encourages nasal breathing; and is under the quite mistaken impression that one pint of ether per hour is needed to maintain anæsthesia by the open method of giving this drug.

The author has succeeded in compressing into a very compact form an enormous amount of instruction and information upon the subject with which he deals.

Altogether the impression gained by perusal of the first two volumes of the London University Manuals is that a most auspicious start has been made.

